# Oncogenic lncRNA ZNFX1 antisense RNA 1 promotes osteosarcoma cells proliferation and metastasis by stabilizing serine and arginine‑rich splicing factor 3

**DOI:** 10.1080/21655979.2022.2036900

**Published:** 2022-02-19

**Authors:** Yang Zhang, Wenbo Xu, Yanlong Wang, Jianming Li, Guanyi He, Mingyan Guan, Xiangyu Zeng, Wei Bian, Yan Song, Jianyu Liu

**Affiliations:** a Department of Orthopaedics, The Second Affiliated Hospital of Harbin Medical University, Harbin, P. R. China; b Department of Operating Room, The Second Affiliated Hospital of Harbin Medical University, Harbin, P. R. China

**Keywords:** Osteosarcoma, ZFAS1, SRSF3, cancer proliferation, metastasis, biomarker

## Abstract

Recent studies have demonstrated that lncRNAs play an important role in cancers, particularly osteosarcoma. ZFAS1 is a newly identified and characterized lncRNA linked to a variety of cancers. The role of ZFAS1 in osteosarcoma is mainly unknown. This study discovered that ZFAS1 was upregulated in osteosarcoma patient tissues, which correlates with elevated SRSF3 protein levels. Higher levels of ZFAS1 or SRSF3 were linked to a poor prognosis of osteosarcoma. ZFAS1 knockdown decreased SRSF3 protein levels but had a negligible effect on SRSF3 mRNA expression. Further research indicated that ZFAS1 could bind to the SRSF3 protein directly and prevent degrading. Functional studies revealed that ZFAS1 knockdown inhibited osteosarcoma cell proliferation as measured by the CCK-8 assay, colony formation assay, and Ki-67 immunofluorescence staining. Furthermore, ZFAS1 knockdown reduced the expression of PCNA, CDK1, CDK4, and CDK6, increasing p53 and p16. IT has also been observed that ZFAS1 knockdown inhibited osteosarcoma cell migration and invasion as measured by the wound healing assay and the trans-well assay with or without Matrigel.

Furthermore, exogenous SRSF3 expression in ZFAS1-depleted osteosarcoma cells restored SRSF3 expression while simultaneously inhibiting cell proliferation and metastasis. Our findings show that ZFAS1 plays an essential role in osteosarcoma progression by stabilizing the SRSF3 protein. Our study provides novel insight into the role of ZFAS1 in osteosarcoma. ZFAS1 has the potential to be used as a prognostic biomarker as well as a therapeutic target in the treatment of osteosarcoma.

## Graphical Abstract

**Figure:**
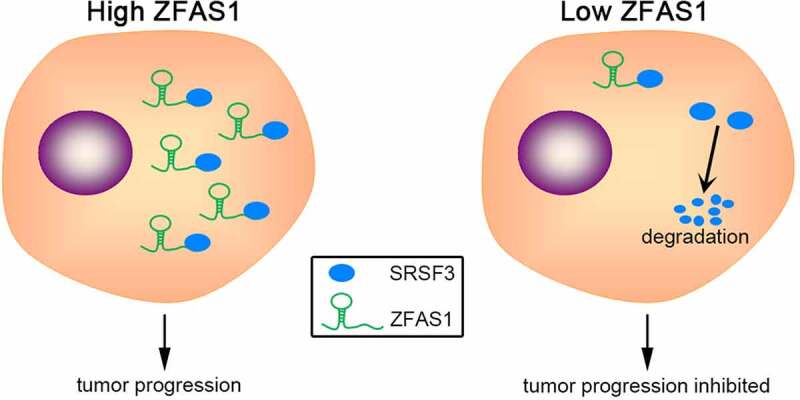

## Introduction

Osteosarcoma is the most common type of malignant bone tumor, primarily affecting children and young adults [1]. Several advanced therapeutic strategies have been developed in recent decades, but many patients still face a high risk of recurrence or unresectable metastasis after treatment [2]. The overall survival rate of osteosarcoma patients remains low [3,4]. Exploration of the molecular mechanisms underlying osteosarcoma initiation and progression and the identification of new prognostic biomarkers is becoming increasingly important.

Long non-coding RNAs (lncRNAs) are RNA transcripts longer than 200 nucleotides lacking protein-coding capability [5]. LncRNAs have been linked to various diseases [6–8], especially in different cancers [9,10]. LncRNAs typically function as post-transcriptional epigenetic regulators of miRNA absorption and protein binding [11]. Numerous lncRNAs have been found in the published literature to be abnormally expressed in osteosarcoma [12]. This aberrant expression can be demonstrated by lncRNA DANCR promoting ROCK1-mediated proliferation and metastasis by decoying miR-335-5p and miR-1972 [13]. EPIC1 inhibits osteosarcoma progression by promoting MEF2D ubiquitylation [14]. ITGB2-AS1 promotes osteosarcoma cells proliferation and metastasis through Wnt/β-catenin signaling [15]. lncRNAs are involved in all osteosarcoma aspects [16,17]. Contemporary research is conducted to uncover the biological function of osteosarcoma-associated lncRNAs.

ZNFX1 antisense RNA 1 (ZFAS1) is a newly discovered lncRNA that plays an oncogenic role in various cancers [18,19]. Recent studies have demonstrated an elevated expression of ZFAS1 in osteosarcoma [20]. Silencing of ZFAS1 represses osteosarcoma cells proliferation and metastasis, and induces cell apoptosis [21]. However, more research into ZFAS1-regulated molecular mechanisms is required.

Based on the bioinformatic analysis, ZFAS1 may directly bind to an important alternative RNA splicing factor, serine and arginine-rich splicing factor 3 (SRSF3). Thus, we hypothesized that ZFAS1 has the potential to regulate osteosarcoma progression through directly binding to and stabilizing SRSF3. ZFAS1 was found to be upregulated in osteosarcoma and be positively correlated with SRSF3 protein levels. Higher levels of ZFAS1 and SRSF3 were associated with a poor prognosis. Further research revealed that ZFAS1 could bind to and stabilize SRSF3 protein, thereby maintaining a high SRSF3 protein level in SRSF3. Functional studies demonstrated that ZFAS1 depletion could inhibit osteosarcoma cells proliferation and metastasis through SRSF3. Our findings suggest that ZFAS1 plays a pro-oncogenic role in osteosarcoma and can be employed as a biomarker and therapeutic target in the disease treatment.

## Methods and reagents

### Collection of osteosarcoma patient tissues

Surgically resected osteosarcoma patient tissues and matched adjacent normal specimens were collected from ten osteosarcoma cases at Harbin Medical University’s Second Affiliated Hospital (Harbin, China). The Ethics Committee approved this study of Harbin Medical University. Every patient provided informed consent for tissue collection. All tissue samples were immediately frozen and preserved in liquid nitrogen until used in this study.

### Cell culture

U2OS human osteosarcoma cells were purchased from the Cell Bank of the Chinese Academy of Sciences (Shanghai, China). Cells were cultured in Dulbecco’s modified Eagle medium (Invitrogen, Carlsbad, CA, USA) contained 10% fetal bovine serum (FBS; GIBCO, USA), 100 μg/mL streptomycin, and 100 U/mL penicillin G (GIBCO). The cells were incubated in the humid incubator under 37°C and 5% CO_2_ conditions.

### Cell transfection

The ZFAS1 siRNA (GCGUGAACUCCUGAGGCGAUU) and negative control siRNA (CGUACGCGGAAUACUUCGAUU) were synthesized by Sangon Biotech, Shanghai, China. SRSF3 expression vector was purchased from GeneCopoeia, Guangzhou, China.

U2OS cells were seeded into 6 cm dishes 24 h before transfection for adherence. Then, indicated control siRNA or ZFAS1 siRNA or/and SRSF3 expression vector were transfected using lipofectamine 2000 (Invitrogen) with serum-free medium. Five hours later, cells were transferred to a complete medium. Cell lysates were harvested 48 h after transfection.

### RNA extraction and real-time PCR

Cells were lysed, and total RNA was extracted using TRIzol reagent (Invitrogen). RNA concentrations were measured using nanodrop. 1 μg total RNA was reverse transcribed into cDNA using high-capacity cDNA reverse transcription kit (Thermo Fisher, Waltham, MA). Real-time PCR was performed using Power SYBR Green PCR Master Mix (Thermo Fisher Scientific, Waltham, USA). PCR conditions were 35 cycles of 94°C for 20seconds, 60°C for 20s, and 72°C for 20s. Relative gene expression levels were calculated by the 2^−ΔΔCt^ method.

The following primer sets were used to measure ZFAS1 and SRSF3 expression:

ZFAS1-F: 5’- AACCAGGCTTTGATTGAACC −3’; ZFAS1-R: 5’- ATTCCATCGCCAGTTTCT −3’ SRSF3-F: 5’- ATGGAAGAACACTATGTGGCTG −3’; SRSF3-R: 5’- GGGACGGCTTGTGATTTCTCT −3’ β-actin-F: 5’- CATGTACGTTGCTATCCAGGC −3’; β-actin-R: 5’- CTCCTTAATGTCACGCACGAT −3’. β-actin was used for normalization.

### Antibodies and western blotting

Cells were lysed with RIPA lysis buffer containing protease inhibitor cocktail (Roche, Switzerland). The protein concentration was determined using the Bradford method (Bio-Rad, Hercules, CA, USA). Equal amounts of proteins were separated by SDS-PAGE and transferred to the nitrocellulose membrane. After blocking with 5% skim milk, the blots were probed with primary antibodies to SRSF3, PCNA, CDK4, CDK6 (Cell signaling technology, USA), CDK1, p53, p16, and actin (Santa Cruz, USA). After washing and incubating with rabbit or mouse secondary antibodies (Cell signaling, USA), the blots were visualized by ECL reagent (GE Healthcare, USA).

### CCK-8 cell viability assay

A CCK-8 assay was used to detect cell viability, as previously described [22]. U2OS cells transfected with ZFAS1 siRNA or control siRNA were seeded into 96-well plates (3 × 10^3^ cells per well) followed by 4-day culture. Cell viability was determined using the Cell Counting Kit-8 (Dojindo, Japan) on days 0, 2, and 4.

### Colony formation assay

A colony formation assay was used to detect cell growth, as previously described [23]. 8 × 10^2^ U2OS cells transfected with ZFAS1 siRNA or control siRNA were counted and seeded into 6 cm dishes. After 12 days of culturing, colonies were stained with 0.1% crystal violet in 20% methanol for 20 min. The dishes were photographed, and the visible colonies were counted.

### Ki-67 immunofluorescence staining

As previously described, a colony formation assay was used to detect cell proliferation capability [22]. U2OS cells were seeded on coverslips and transfected with ZFAS1 siRNA or control siRNA. After 48 h of transfection, cells were fixed with 4% PFA. Consequently, fixed cells were incubated with Ki-67 antibody (Cell Signaling Technology, USA) for one hour, followed by 20 min of incubation at room temperature. Cells were then counterstained with DAPI for the cell nucleus. All coverslips were mounted using a prolong® diamond antifade mountant (Applied Biosystems, USA).

### Wound-healing assay

As previously described, a wound-healing assay was used to detect cell migration [22]. U2OS cells transfected with ZFAS1 siRNA or control siRNA were seeded in 3.5 cm dishes and grown to a density of 80%. Artificial wounds were created using a 200-μL pipette tip. The migration distance was determined 24 h after scratching.

### Trans-well assay

Trans-well assay was used to detect cell migration and invasion, as per the prescribed protocol [22].

For migration analysis, U2OS cells transfected with ZFAS1 siRNA or control siRNA were seeded in a 24-well transwell unit (5 × 10^4^ cells/well) with 8-μm polycarbonate nucleopore filters (Corning Costar, Corning, NY, USA). The upper compartment contained a serum-free medium, whereas the lower compartment contained a medium containing 10% FBS. Cells were then incubated for 24 h in a humid incubator under 37°C and 5% CO_2_ conditions. Crystal violet staining was used to fix and count cells adhering to the lower surface of the filter.

For the invasion assay, the membrane of the trans-well unit was coated with 40 μL Matrigel (BD Biosciences, USA) and incubated at 37°C for 4 h to form a reconstructed basement membrane. The cells were examined using the same techniques as in the migration assay.

### Xenograft tumor experiments

Athymic BALB/c nude mice (6 ~ 8 weeks old) were purchased from Shanghai Model Organisms Center, Inc., Shanghai, China. All mice were housed under specific pathogen-free conditions.

3 × 10^6^ of U2OS cells transfected with indicated reagents were subcutaneously injected into the right shank of nude mice. The tumors’ maximum (L) and minimum (W) lengths were measured using a caliper every week. The tumor size was calculated as ½LW^2^. Three weeks later, all mice were sacrificed, and the xenografts were isolated and weighed.

### Statistical analysis

Data from at least three individual experiments were presented as mean ± SD. Statistical data were analyzed using Statistical Program for Social Sciences (SPSS) 17.0 software (SPSS, Chicago, IL, USA). The student’s t-test was implemented for comparison. P < 0.05 represented statistical significance.

## Results

We hypothesized in our study that the lncRNA ZFAS1 promotes osteosarcoma by directly binding to and stabilizing SRSF3. First, we examined the expressions of ZFAS1 and SRSF3 in osteosarcoma patients and their relationship to prognosis outcomes. Subsequently, the interaction between ZFAS1 and SRSF3 was tested, and the SRSF3 protein stability was identified upon ZFAS1 deficiency. Moreover, we demonstrated the biological effects of ZFAS1 on cell proliferation and metastasis in osteosarcoma cell lines U2OS cells and MG-63 cells. In addition, we evaluated the impact of ZFAS1 on osteosarcoma progression in vivo.

### Both ZFAS1 and SRSF3 are over-expressed in osteosarcoma and indicate a poor prognosis

**Figure 1.**
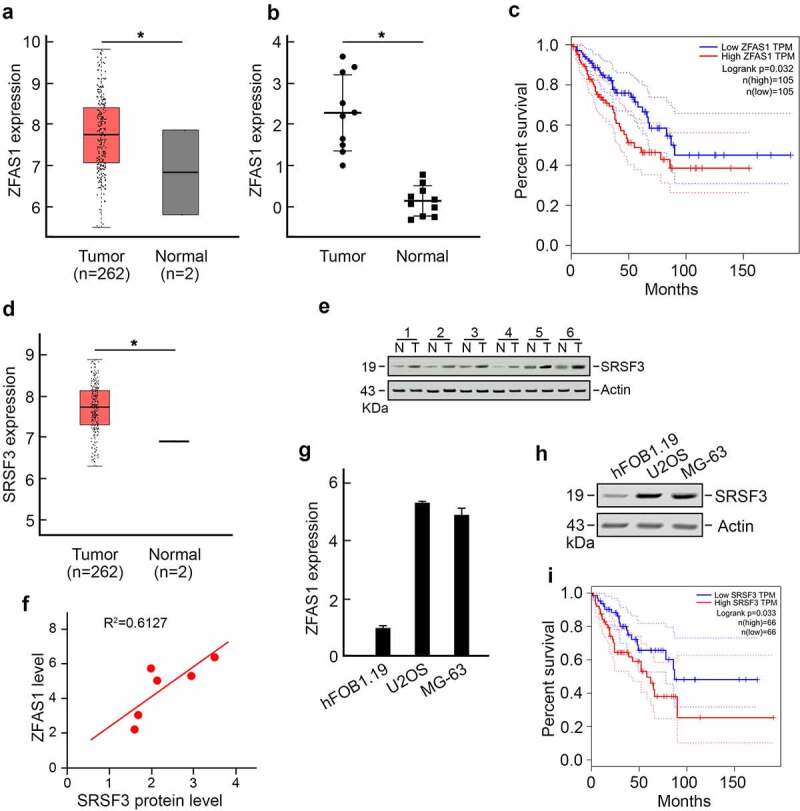
Increased level of ZFAS1 is positively correlated with elevated SRSF3 protein level and indicates a worse prognosis of osteosarcoma.

The role of ZFAS1 in osteosarcoma is investigated in this study. ZFAS1 was found to be significantly overexpressed in osteosarcoma patient samples, according to data from the TCGA database (Figure 1a). Following that, we discovered that ZFAS1 levels were substantially higher in 10 of our collected osteosarcoma tissues when compared to matched adjacent normal tissues. We also found that ZFAS1 levels were considerably higher in 10 of our collected osteosarcoma tissues when compared to matched adjacent normal tissues (Figure 1b). In addition, Kaplan-Meier analysis showed that a higher level of ZFAS1 indicated significantly shorter overall survival of osteosarcoma patients (Figure 1c).

**(a**) ZFAS1 level was upregulated in osteosarcoma. Data from TCGA database. *p < 0.05. **(b)** ZFAS1 level was upregulated in osteosarcoma patient tissues compared to adjacent normal tissues. *p < 0.05. **(c)** Higher ZFAS1 levels indicated a poor prognosis of osteosarcoma. **(d)** SRSF3 expression was increased in osteosarcoma. Data from TCGA database. *p < 0.05. **(e)** SRSF3 protein level was upregulated in osteosarcoma patient tissues compared to adjacent normal tissues. Actin was served as the loading control. **(f)** ZFAS1 level was positively correlated with the SRSF3 protein level. **(g)** ZFAS1 expression in osteosarcoma cells compared to normal hFOB1.19 normal human osteoblast cells. **(h)** SRSF3 expression in osteosarcoma cells compared to normal hFOB1.19 normal human osteoblast cells. **(I)** Higher SRSF3 levels indicated a poor prognosis of osteosarcoma.

Interestingly, we discovered that a major splicing factor, serine, and arginine-rich splicing factor 3 (SRSF3), was also upregulated in osteosarcoma (Figure 1d) using TCGA datasets. An increased level of SRSF3 was detected in osteosarcoma tissue (Figure 1e). Pearson correlation analysis revealed a strong positive correlation between ZFAS1 level and SRSF3 protein level (figure 1f). These findings were also observed in U2OS cells and MG-63 osteosarcoma cell lines compared to hFOB1.19 normal human osteoblast cell line. U2OS cells and MG-63 cells harbored significantly higher levels of ZFAS1 and SRSF3 compared to hFOB1.19 cells (Figure 1g, H). Furthermore, higher levels of SRSF3 were associated with significantly shorter overall survival in osteosarcoma patients (Figure 1i).

### ZFAS1 stabilizes SRSF3 protein in osteosarcoma

**Figure 2.**
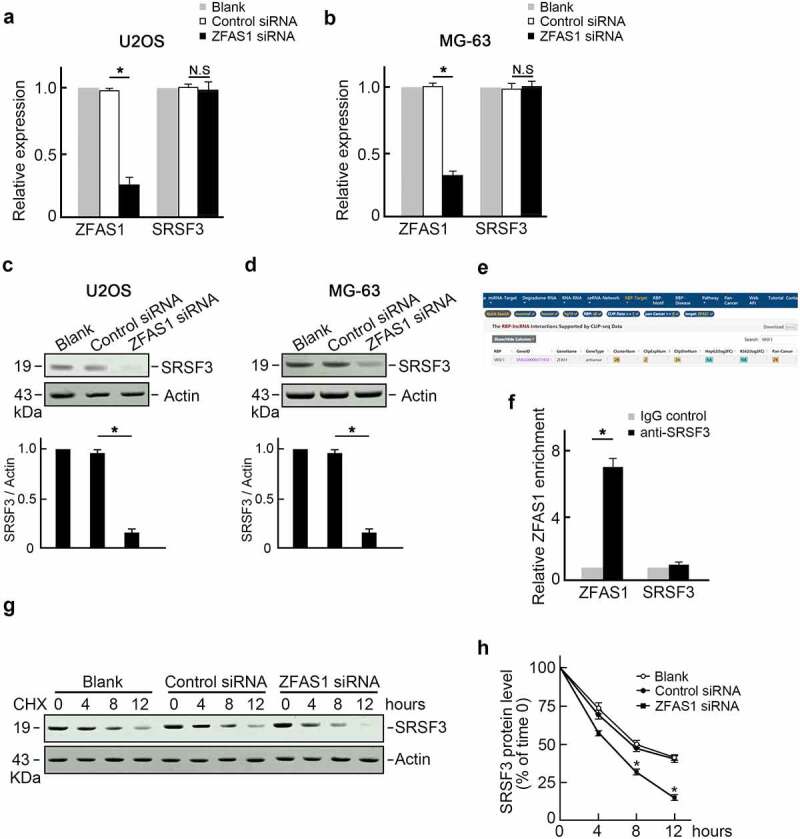
ZFAS1 directly binds to and stabilizes SRSF3 protein.

Speculation regarding ZFAS1 regulation of SRSF3 expression was positive correlations with SRSF3 levels. This speculation was addressed through the transfection of U2OS cells with ZFAS1 siRNA, and the level of SRSF3 was measured (Figure 2a). Surprisingly, knocking out ZFAS1 reduced SRSF3 protein levels (Figure 2b) but did not affect SRSF3 mRNA levels (Figure 2a). This finding suggested that ZFAS1 could stabilize SRSF3 protein and influence ZEB2 degradation. Thus, we analyzed potential interaction between ZFAS1 and SRSF3 proteins using The Encyclopedia of RNA Interactomes (ENCORI) database. SRSF3 was identified as a potential ZFAS1 binding protein (Figure 2c). RNA-binding protein immunoprecipitation (RIP) assay using SRSF3 antibody demonstrated a remarkable enrichment of endogenous ZFAS1 with SRSF3 protein compared with negative IgG and unrelated GAPDH mRNA (Figure 2d). Furthermore, U2OS cells transfected with ZFAS1 siRNA or control siRNA were treated with cycloheximide (CHX) to inhibit protein biosynthesis. SRSF3 protein levels were reduced more rapidly in ZFAS1 knockdown U2OS cells, indicating that SRSF3 protein has a shorter half-life after ZFAS1 knockdown. (Figure 2e, f). These findings suggested that ZFAS1 positively regulated SRSF3 levels by stabilizing SRSF3 protein.

**(a-b)** Transfection of ZFAS1 successfully reduced ZFAS1 level but did not alter SRSF3 mRNA level in U2OS cells (A) and MG63 cells (B). n = 3, *p < 0.05. **(c-d)** Knockdown of ZFAS1 reduced SRSF3 protein level in U2OS cells (C) and MG63 cells (D). Actin was served as the loading control. Representative blots were shown (upper panel). Ratios of levels of SRSF3 vs. Actin were calculated after densitometric analysis of blot images using NIH Image J 1.61 (lower panel). n = 3. *p < 0.05. **(e)** Bioinformatics analysis by the ENCORI database predicted SRSF3 was a potential binding protein of ZFAS1. **(f)** The fold enrichment of ZFAS1 in SRSF3 RIP is relative to its matching IgG control RIP. n = 3, *p < 0.05. **(g)** U2OS cells transfected with control siRNA or ZFAS1 siRNA were treated with CHX to inhibit protein biosynthesis. The levels of SRSF3 were examined by Western blot at 0, 4, 8, and 12 h post-treatment. Representative images from three individual experiments were shown. **(h)** knockdown of ZFAS1 resulted in a shorter SRSF3 protein half-life.

### Knockdown of ZFAS1 inhibits osteosarcoma cells proliferation and metastasis

**Figure 3.**
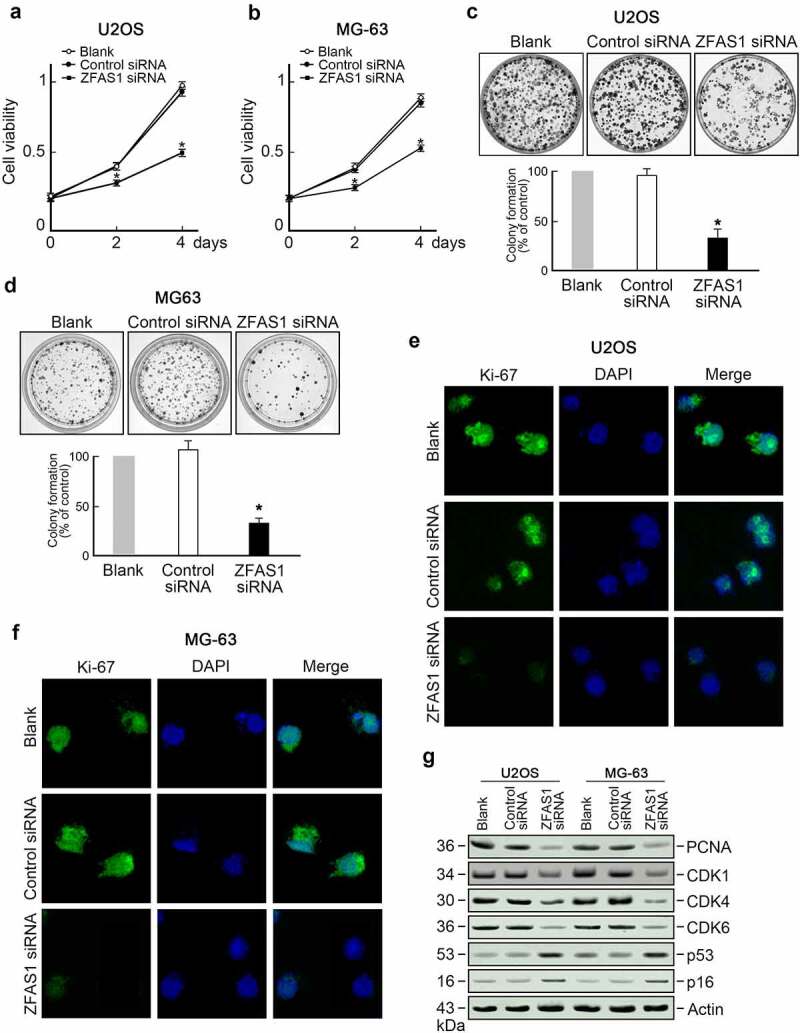
Knockdown of ZFAS1 inhibits osteosarcoma cells proliferation.

The biologic function of ZFAS1 in osteosarcoma cells was explored based on the upregulation of both ZFAS1 and SRSF3 in osteosarcoma. We discovered that knocking out ZFAS1 inhibited the growth of U2OS cells (Figure 3a). Colony formation assay showed fewer colonies were counted in ZFAS1 knockdown groups compared to the control group (Figure 3b). Immunofluorescence staining of Ki-67, a cell proliferation marker, revealed a solid Ki-67 signal in control cells but an exceptionally low Ki-67 signal in ZFAS1 knockdown U2OS cells (Figure 3c). Furthermore, we discovered that the levels of several cell cycle promoting factors, such as PCNA, CDK1, CDK4, and CDK6, were reduced in ZFAS1 depleted U2OS cells. In contrast, the levels of two cell cycle inhibitors, p53 and p16, were increased (Figure 3d). These findings indicate that ZFAS1 deficiency inhibited osteosarcoma cell proliferation.

**(a)** Knockdown of ZFAS1 reduced U2OS cells viability. n = 3, *p < 0.05. **(b)** Knockdown of ZFAS1 reduced MG63 cells viability. n = 3, *p < 0.05. **(c)** Knockdown of ZFAS1 inhibited colony formation of U2OS cells. Representative plates were shown. n = 3, *p < 0.05. **(d)** Knockdown of ZFAS1 inhibited colony formation of MG63 cells. Representative plates were shown. n = 3, *p < 0.05. **(e)** U2OS cells transfected with ZFAS1 siRNA or control siRNA were immunofluorescence stained with Ki-67. Representative images were shown. n = 3. **(f)** MG63 cells transfected with ZFAS1 siRNA or control siRNA were immunofluorescence stained with Ki-67. Representative images were shown. n = 3. **(g)** Levels of PCNA, CDK1, CDK4 CDK6, p53, and p16 in U2OS cells or MG63 cells transfected with ZFAS1 siRNA or control siRNA were examined by immunoblotting. Actin was served as the loading control. Representative blots were shown. n = 3.

**Figure 4.**
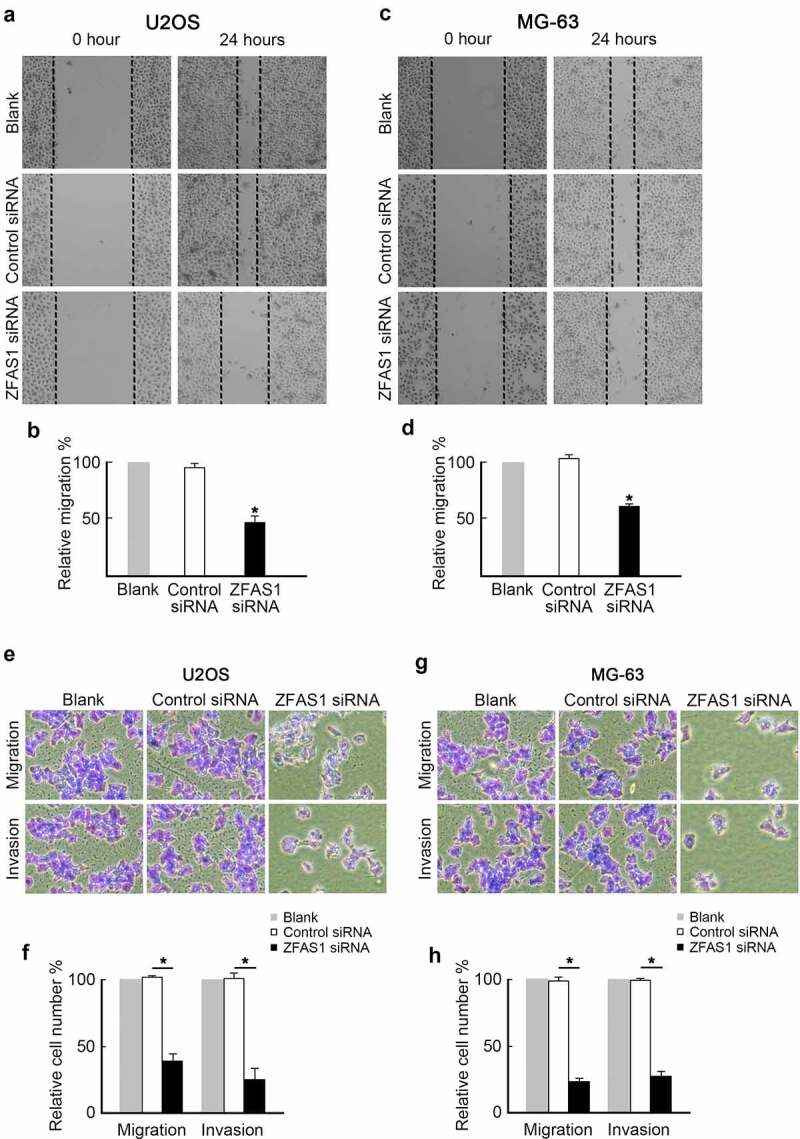
Knockdown of ZFAS1 inhibits osteosarcoma cells metastasis.

The influence of ZFAS1 on osteosarcoma cells metastasis was further investigated. U2OS cells were transfected with ZFAS1 siRNA or control siRNA to establish the impact of ZFAS1 on osteosarcoma cells’ metastasis. Subsequently, U2OS cells were subjected to wound healing and transwell assays. We discovered that ZFAS1 depletion significantly slowed healing compared to control cells (Figure 4a, B). Transwell assay data showed that knockdown of ZFAS1 decreased the migration and invasion capabilities of U2OS cells (Figure 4c, D). These findings suggest that ZFAS1 deficiency inhibited osteosarcoma cell metastasis.

**(a)** U2OS cells transfected with ZFAS1 siRNA or control siRNA were subjected to wound healing assay. Representative images (100×) were shown. **(b)** Knockdown of ZFAS1 decreased wound healing speed in U2OS cells analyzed from (A). n = 3, *p < 0.05. **(c)** MG-63 cells transfected with ZFAS1 siRNA or control siRNA were subjected to wound healing assay. Representative images (100×) were shown. **(d)** Knockdown of ZFAS1 decreased wound healing speed in MG-63 cells analyzed from (A). n = 3, *p < 0.05. **(e)** U2OS cells transfected with ZFAS1 siRNA or control siRNA were subjected to the trans-well assay with or without Matrigel. Representative images (200×) were shown. **(f)** Knockdown of ZFAS1 decreased U2OS cells migration and invasion analyzed from (C). **(g)** MG-63 cells transfected with ZFAS1 siRNA or control siRNA were subjected to the trans-well assay with or without Matrigel. Representative images (200×) were shown. **(h)** Knockdown of ZFAS1 decreased MG-63 cells migration and invasion analyzed from (G).

### ZFAS1 promotes osteosarcoma progression through SRSF3

**Figure 5.**
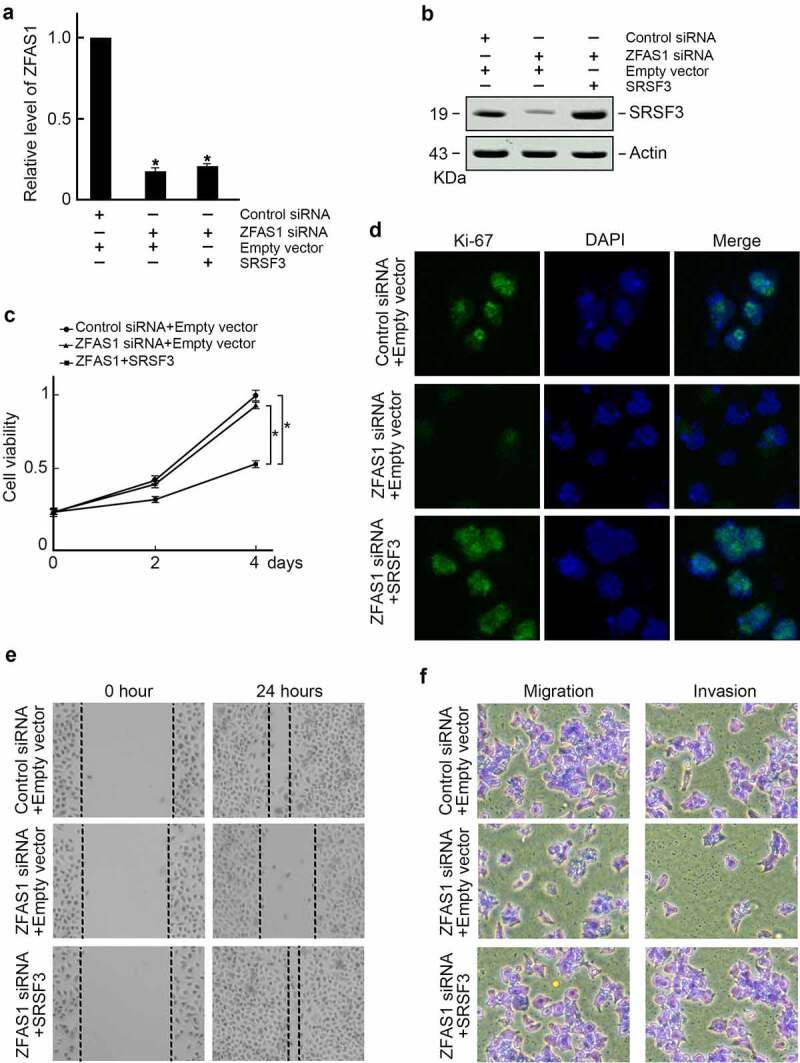
Ectopic expression of SRSF3 restored proliferation and metastasis capabilities in U2OS cells depleted of ZFAS1.

Based on the findings of this study, ZFAS1 stabilized SRSF3 protein in osteosarcoma cells. The role of ZFAS1 regulated osteosarcoma cells proliferation and metastasis through SRSF3 was subsequently explored. As a result, SRSF3 expression vector was co-transfected into ZFAS1 knockout cells (Figure 5a). Immunoblotting confirmed restored SRSF3 protein levels in ZFAS1 knockdown cells (Figure 5b). Exogenous SRSF3 expression reversed the proliferation of U2OS cells inhibited by ZFAS1 knockdown, as measured by the CCK-8 assay (Figure 5c) and Ki-67 immunofluorescence staining (Figure 4d). Exogenous SRSF3 expression also inhibited U2OS cell metastasis, as measured by wound healing assay (Figure 5e) and trans-well assay (figure 5f).

U2OS cells were co-transfected with indicated reagents, and **(a)** Levels of ZFAS1 were examined by qRT-PCR. *p < 0.05; **(b)** Levels of SRSF3 protein were examined by immunoblotting. Actin was served as the loading control. **(c)** Ectopic expression of SRSF3 restored cell growth capabilities in U2OS cells depleted of ZFAS1. *p < 0.05. **(d)** Ectopic expression of SRSF3 restored Ki-67 expression in U2OS cells depleted of ZFAS1. **(e)** Ectopic expression of SRSF3 restored healing speed in ZFAS1 knockdown cells. **(f)** Ectopic expression of SRSF3 reversed cell migration and invasion in ZFAS1 knockdown cells.

**Figure 6.**
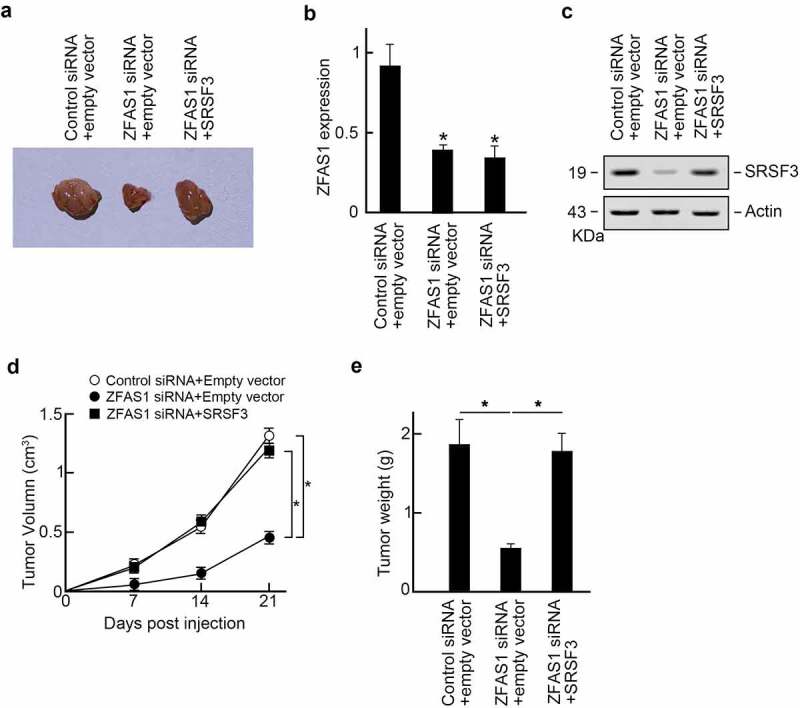
ZFAS1 promotes osteosarcoma progression in vivo through SRSF3.

The following experiment elucidated the role of ZFAS1 promoted osteosarcoma progression in vivo. U2OS cells were transfected with control or ZFAS1 siRNA, and SRSF3 expression vectors were subcutaneously injected into the flanks of the athymic Balb/c nude mice (6 ~ 8 weeks old). The tumor volumes were measured every seven days. Three weeks later, all mice were sacrificed, and xenograft tumors were isolated (Figure 6a). We found that ZFAS1 siRNA suppressed the ZFAS1 level (Figure 6b) and decreased SRSF3 protein levels (Figure 6c). In comparison, co-transfection of SRSF3 expression vector restored SRSF3 protein level in ZFAS1 silenced xenograft tumors (Figure 6c). Besides, we found that ZFAS1 knockdown significantly reduced xenografts sizes and weights compared to control groups, but SRSF3 co-expression reversed tumor progression upon ZFAS1 knockdown (Figure 6d-E). Compilation of these findings suggests that ZFAS1 promotes osteosarcoma progression through SRSF3.

**(a)** Images of xenografts tumors. **(b)** ZFAS1 expression in xenografts. **(c)** SRSF3 protein levels in xenografts. Actin was served as the loading control. **(d and e)** ZFAS1 knockdown reduced tumor volumes (D) and tumor sizes (E), but exogenous SRSF3 expression reversed these phenotypes.

## Discussion

Osteosarcoma is one of the most common cancers that affect the young population [24]. Based on a solid body of evidence, non-coding RNAs, particularly lncRNAs, are essential for osteosarcoma tumorigenesis and progression [25–27]. In our present study, we investigated the biological function of ZFAS1 in osteosarcoma cells proliferation and metastasis and its related molecular mechanism. We found that the expression of ZFAS1 was increased in osteosarcoma clinical tissues. This correlation was consistent with previous research, indicating that ZFAS1 plays an essential role in osteosarcoma.

Several studies have found that ZFAS1 promotes osteosarcoma via ceRNA mechanisms. Through BMI1 and ZEB2, ZFAS1 regulates osteosarcoma cell growth and metastasis [28]. ZFAS1 promotes tumorigenesis in osteosarcoma through miR-646/NOB1 [29]. Distinguished from previous research, we demonstrated that ZFAS1 could directly bind to the critical pro-oncogenic factor SRSF3. This interaction promotes osteosarcoma progression by preventing SRSF3 degradation.

SRSF3, also known as SRp20, is a serine-arginine-rich splicing factor critical for pre-mRNA splicing and mRNA stability. Recent research has identified SRSF3 as a proto-oncogene. SRSF3 regulated the expressions or splicing events of nearly 200 genes in osteosarcoma cells based on global profiling data [30]. These genes, such as ANXA1, SMC2, KIF23, and DDX5, are primarily associated with cancer cell proliferation, metastasis, and poor prognosis [31–34]. SRSF3 was discovered to be overexpressed in osteosarcoma. The exact mechanism by which SRSF3 is increased in osteosarcoma remains unknown. We discovered that SRSF3 was increased in osteosarcoma tissues, consistent with previous research.

Furthermore, increased SRSF3 levels were found to be positively correlated with ZFAS1. Further functional experiments revealed that ZFAS1 could stabilize SRSF3 protein, increasing its level in osteosarcoma cells. To the best of our knowledge, this is the first study to provide evidence for the mechanism of SRSF3 upregulation in osteosarcoma.

## Conclusions

Based on the findings of this study, the role of ZFAS1 in osteosarcoma cells proliferation and metastasis was established through the stabilization of alternative splicing factor SRSF6. These findings provide a novel insight into ZFAS1 function and SRSF3 up-regulation in osteosarcoma progression, enabling ZFAS1 to become a potential target for osteosarcoma treatment.

## Data Availability

The data supporting this study’s findings are available from the corresponding author upon reasonable request.

## Highlight

1. Both ZFAS1 and SRSF3 are up-regulated in osteosarcoma and are associated with poor prognosis.
2. ZFAS1 directly binds to SRSF3 protein to prevent degradation.
3. ZFAS1 promotes osteosarcoma cells proliferation and metastasis through SRSF3.
